# Assessing multi-decadal land-cover – land-use change in two wildlife protected areas in Tanzania using Landsat imagery

**DOI:** 10.1371/journal.pone.0185468

**Published:** 2017-09-28

**Authors:** Devolent T. Mtui, Christopher A. Lepczyk, Qi Chen, Tomoaki Miura, Linda J. Cox

**Affiliations:** 1 Department of Natural Resources and Environmental Management, University of Hawai‘i at Mānoa, Honolulu, United States of America; 2 Department of Geography, University of Hawai‘i at Mānoa, Honolulu, United States of America; Bristol University/Remote Sensing Solutions Inc., UNITED STATES

## Abstract

Landscape change in and around protected areas is of concern worldwide given the potential impacts of such change on biodiversity. Given such impacts, we sought to understand the extent of changes in different land-cover types at two protected areas, Tarangire and Katavi National Parks in Tanzania, over the past 27 years. Using Maximum Likelihood classification procedures we derived eight land-cover classes from Landsat TM and ETM+ images, including: woody savannah, savannah, grassland, open and closed shrubland, swamp and water, and bare land. We determined the extent and direction of changes for all land-cover classes using a post-classification comparison technique. The results show declines in woody savannah and increases in barren land and swamps inside and outside Tarangire National Park and increases in woody savannah and savannah, and declines of shrubland and grassland inside and outside Katavi National Park. The decrease of woody savannah was partially due to its conversion into grassland and barren land, possibly caused by human encroachment by cultivation and livestock. Based upon these changes, we recommend management actions to prevent detrimental effects on wildlife populations.

## Introduction

Changes in quality and quantity of land-cover–land-use (LCLU) have implications for the continual existence of wildlife species [1,2]. In semiarid African countries, LCLU change is driven by precipitation [3–6], fire [7–10], high densities of large herbivores when their movement is restricted [11], [4], fire [12,13], people [8], and through a combination of these variables [14] [4], [8]. In these semiarid systems a mean annual rainfall above 650 mm promotes existence of closed canopy trees, whereas at about 650 mm results in a co-existence of woody and grass cover, while below 350 mm allows existence of large patches of grassland, and quite low woody cover [3], [15]. Furthermore, frequent fires tend to depress woody cover [4]. Hence, rainfall and fire, in combination with intense browsing and grazing by wildlife species, drive vegetation dynamics in these systems [4], [7], [11,12], [16]. For instance, the interaction of rainfall and herbivory may lead to the conversion of grassland to woodland or woodland to grassland [3], [13]. Low rainfall reduces the availability of the grazing resource, causing overgrazing which suppresses fire intensity possible, thus reducing tree damage, and the consequent conversion of grassland or savannah into woody savanna [8], [12]. Excessive browsing stimulates grass growth, increasing fuel load and therefore intensifying fires that convert woody savanna to savanna or grassland [13] and [12]. Human settlers directly affect land-cover by clearing land for cultivation and harvesting trees or grasses, and may initiate fires [8], that reduce dispersal areas for migratory species [17–19], and block wildlife migratory routes [20,21], resulting in declining wildlife populations [22].

One strategy used worldwide to prevent degradation of wildlife habitats and ensure the long-term survival of wildlife species is to create protected areas (PAs), such as national parks. The effectiveness of PAs is frequently questioned in developing countries because they often adjoin poor communities that rely on wildlife resources and their habitats to sustain their livelihoods [23,24]. Nevertheless, PAs have been found to be effective at decreasing land clearing, logging, and grazing inside the PAs, compared to areas outside their boundaries [25–29].

Land-cover-land-use change have affected wildlife species in and around PAs in Tanzania, due to the establishment and expansion of villages and changes in agricultural policies that were established in 1974 and 1983 to improve social welfare (Prins 1987). These policies increased land degradation via increased settlements, livestock herds, farming, and mining [30], [20], [31]. For example, between 1957 and 1987, 77% (from 630 km^2^ to 144 km^2^) of woody vegetation within a distance of less than 40 km from the Tarangire National Park (TNP) boundary in the Masai Steppe was converted into grassland and cultivated farms [20]. As a result, four of the nine wildlife corridors on the western and southern sides of TNP were blocked, causing the wildebeest population to decrease from 40,000 to 5,000 [31]. Furthermore, human migrants from northern Tanzania settled in the open area south of the Katavi National Park (KNP) in the late 1990, with their cattle grazing inside the protected area (Tim Caro, pers. comm.). In the northwest of KNP the flow of Katuma River has been reduced due to upstream rice cultivation, reducing the water available to maintain the wetlands of Lakes Chada and Katavi and the Katisunga flood plain, which harbour the highest animal density in the park during dry seasons [32,33]. These activities all have notable effects on the types of land-cover utilized by wildlife species, and ultimately on these species populations. However, no recent evaluation of land-cover change in the TNP and KNP ecosystems has been conducted.

In this study we use Landsat Thematic Mapper (TM) and Enhanced Thematic Mapper plus (ETM^+^) imagery to identify and quantify LCLU change in wildlife PAs in Tanzania. Several studies conducted in Tanzania have used this approach, including Msoffe and others [31] who used Landsat (TM and ETM^+^) images of 1984 and 2000 to assess land-use changes in the Tarangire wildlife ecosystem, and Pelkey and others [34] who used Advanced Very High Resolution Radiometer (AVHRR) imagery of 1982 and 1994 to assess habitat changes in terms of greenness, in PAs across Tanzania. The advantage of these image analyses is that they provide a powerful tool to understand LCLU change at large physical extents in and around PAs and the likely impact it has on sustaining wildlife species. Thus, our goal was to understand the extent to which changes have taken place in the different types of land covers that could be potentially detrimental to wildlife conservation, inside and outside two PAs in Tanzania. We sought to address two main questions. First, to what extent have the areas of different types of land-cover inside and outside the national parks changed over the past 27 years? Second, were the proportions of changes in each type of land-cover similar inside and outside the parks?

## Methods

**Fig 1:**
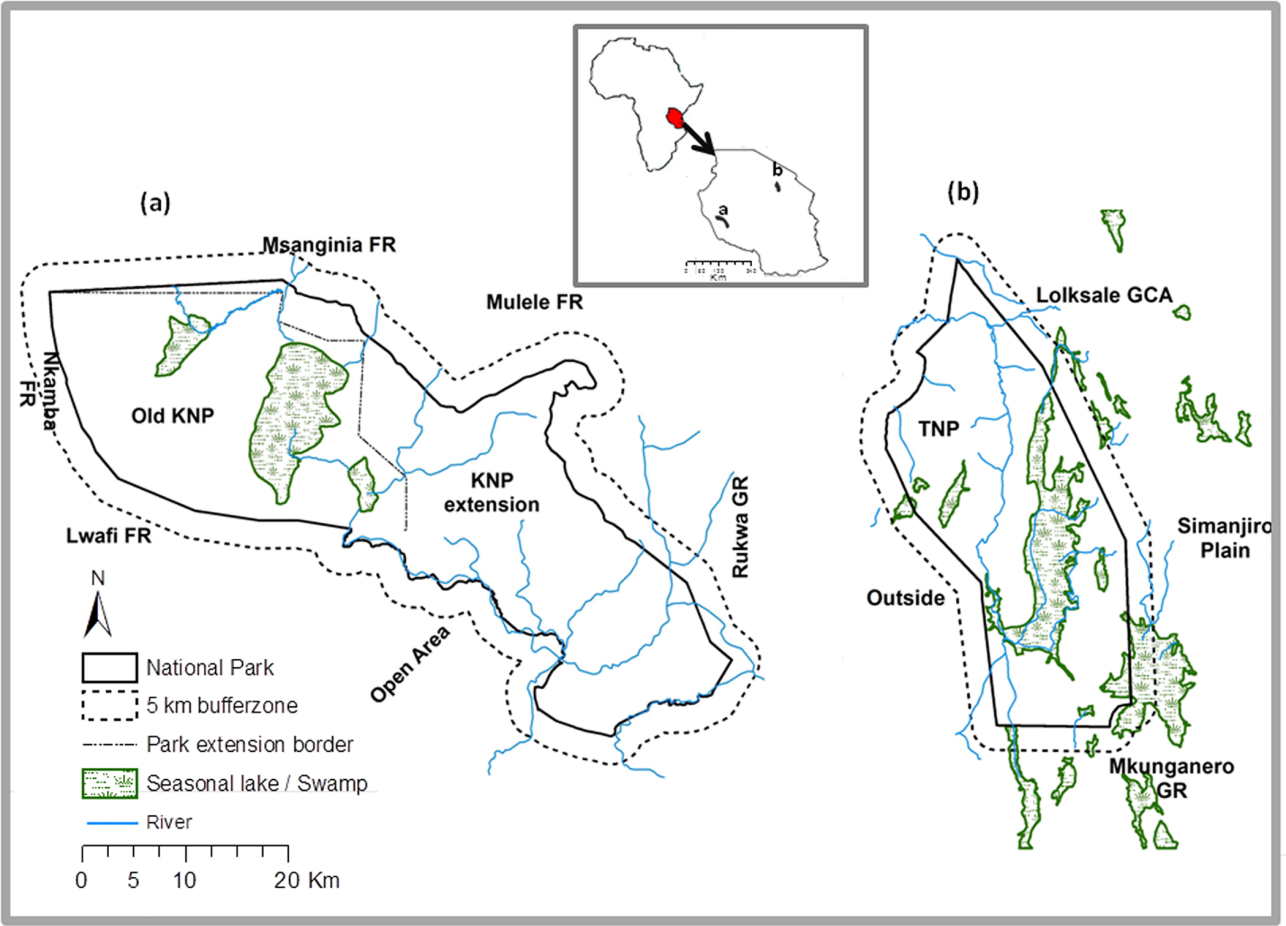
Inset map (i) of Tanzania (ii), and positions of Katavi (iii) and Tarangire (iv) National Parks in Tanzania. The Katavi map shows the main Katuma river, which flows from north west towards south east, the major swamps that harbor high density of large mammals, particularly during dry seasons, and the adjacent areas. The Tarangire map also shows the major swamps, the Tarangire River, and adjacent areas bordering the park.

Land-cover changes were investigated for the TNP and KNP in Tanzania (Fig 1). The TNP covers 2,600 km^2^ (between 3^o^40^’^ to 5^o^35^’^ latitude, and 35^o^45^’^ to 37^o^longitude) at an elevation ranging from 1,200 m to 1,600 m above sea level. The park was established as a Game Reserve in 1957 and declared a National Park in 1970. The TNP is bordered by Lake Natron and the Mto-wa-Mbu Game Controlled Area (GCA) to the north, the Lolkisale GCA and the Simanjiro Plains to the east, the Mkungunero Game Reserve (GR) to the south, and Lake Burunge, the Burunge GCA, the Kwakuchinja Open Area, and Lake Manyara National Park to the west (Fig 1). Average annual rainfall is about 655 mm with short rains between October and December and heavy rains between February and April or May [35]. Temperatures range from 17 ^o^C to 29 ^o^C during the hot season (December to February), and 14 ^o^C to 25 ^o^C in the cold season (June and July) [36]. The major types of vegetation in this park are riparian woodland, wetlands and seasonal flood plain, *Acacia-Commiphora* woodland, riverine grassland, *Combretum-Dalbergia* woodland, *Acacia drepanolobium* woodland, and grasslands with scattered baobab trees (TANAPA *no date*). Pastoralism and agricultural are land use activities practiced around the park [20], [30].

The KNP covers 4,238 km^2^ (between 6°63^’^ to 7°30^’^ latitude, and 30°75’ to 31°74^’^ longitude) at an elevation ranging from 800 m to 1,600 m above sea level. The park was established in 1974 with an area of 1,816 km^2^, but was enlarged in 1998 to reduce pressure from settlements and grazing by cattle. The KNP is bordered by Msanginia Forest Reserve (FR) and Mlele GCA to the north, Lwafi GR and Nkamba FR to the west, Usevya Open Area to the south, and Rukwa GR to the south and south east. Land use activities around the park are horticultural production and pastoralism [32]. Average annual rainfall is about 955 mm, which falls between November and April or May. The vegetation consists of grassland interspersed with miombo woodlands and mixed woodlands. Miombo forms a single story, with a light, closed canopy of deciduous woodland usually greater than 15 m tall dominated by trees of the genera *Brachystegia*, *Julbernadia*, and *Isoberlinia* [7,8], [37]. Underneath the trees are layers of scattered shrubs, grasses, and forbs that grow to a height of 0.3 to 100 cm with 50–75% ground cover [16]. The genera *Markhamia*, *Grewia*, *Terminalia*, *Combretum*, *Syzygium*, and *Acacia* also occur in the miombo woodland of the KNP [38]. During the dry season, large mammals feed on swampy vegetation occurring in the large seasonal lakes of Katavi and Chada, and the Katisunga flood plain [32], which is maintained by the Katuma River (Fig 1).

To address the main research question, land cover was evaluated inside the TNP and KNP and within a 5 km buffer zone around them (Fig 1). Similar studies conducted in the same areas have used a buffer of 10 km [25], but we used a 5 km buffer due to a lack of ground truthing data for land cover-land use to classify satellite images beyond this distance. Parks and the surrounding buffer area may be assumed to have similar physical and environmental conditions, but the existence of heterogeneous land cover can violate this assumption [39] and bias our assessment. Considering the less strict levels of protection on the lands adjoining parks, we assumes that fewer anthropogenic activities occurred inside the parks than in the surrounding areas [25,26], [28].

**Fig 2:**
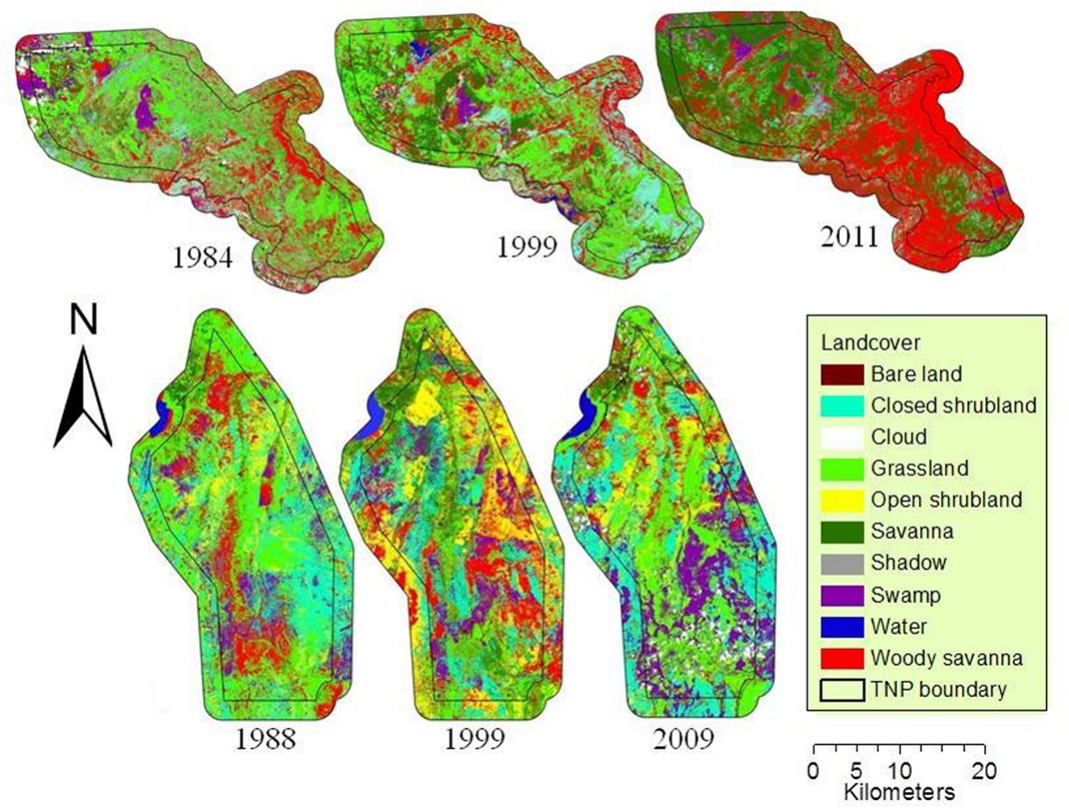
Land-cover maps of Katavi (upper row) and Tarangire (lower row) national parks and their buffer areas derived from the Landsat imagery of 1980s, 1990s and 2010s.

Landsat images were selected to assess land-cover change in the two PAs (Fig 2). The 1988, 1999, and 2009 images (path 168, row 63) were selected for TNP and the 1984, 1999, and 2011 images (path 171, row 65) for KNP. The number of images selected for each park was limited by the availability of cloud-free imagery data for the selected time period. Specifically, we used Thematic Mapper (TM) and Enhanced Thematic Mapper plus (ETM+) images during a period of short rains on 10 Jan 1988, 11 Jan 1999, and 11 Apr 2009 for TNP and during the dry season on 29 Jun 1984, 3 Sep 1999, and 26 Jul 2011 for KNP. The use of data obtained on the same dates is necessary when analyzing remote sensing data because it minimizes change detection errors arising from seasonal differences such as vegetation phenology and sun angle effects [40,41]. All images were obtained from the U.S. Geological Survey (USGS) (https://earthexplorer.usgs.gov/ and https://glovis.usgs.gov/).

**Fig 3:**
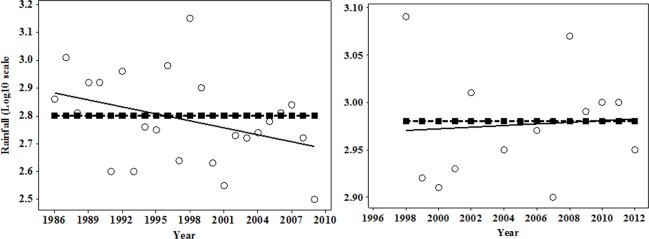
Patterns of total annual rainfall and average annual rainfall for (a) Katavi and (b) Tarangire National Parks. Open circles represent rainfall values and closed squares connected by a broken line represent average annual rainfall values. Annual rainfall did not significantly change over time in Katavi [F_1_, _12_ = 0.09, p = 0.76] or Tarangire [F_1_, _15_ = 2.17, p = 0.16] National Parks [Data source: Tanzania national Parks 2012].

To ensure that assessment of the selected Landsat images of 1980s, 1990s, and 2010s would not provide biased information, we obtained the available rainfall data records for each park from the 1980s to the 2010s (Fig 3). This information was necessary because it is known that vegetation cover in semiarid countries is limited by rainfall [3]. The data were all available for TNP (i.e. 1988, 1999 and 2009), while for KNP rainfall data record for 1984 was missing with 1997 being the first year data was available. In this case we observed NDVI secondary information [34] that was available based on the positive relationship of rainfall and NDVI [5]. According to Pelkey and others [34] the NDVI increased between 1982 and 1994 in some parts of the country including the KNP ecosystem, indicating an increase in rainfall during that period.

**Fig 4:**
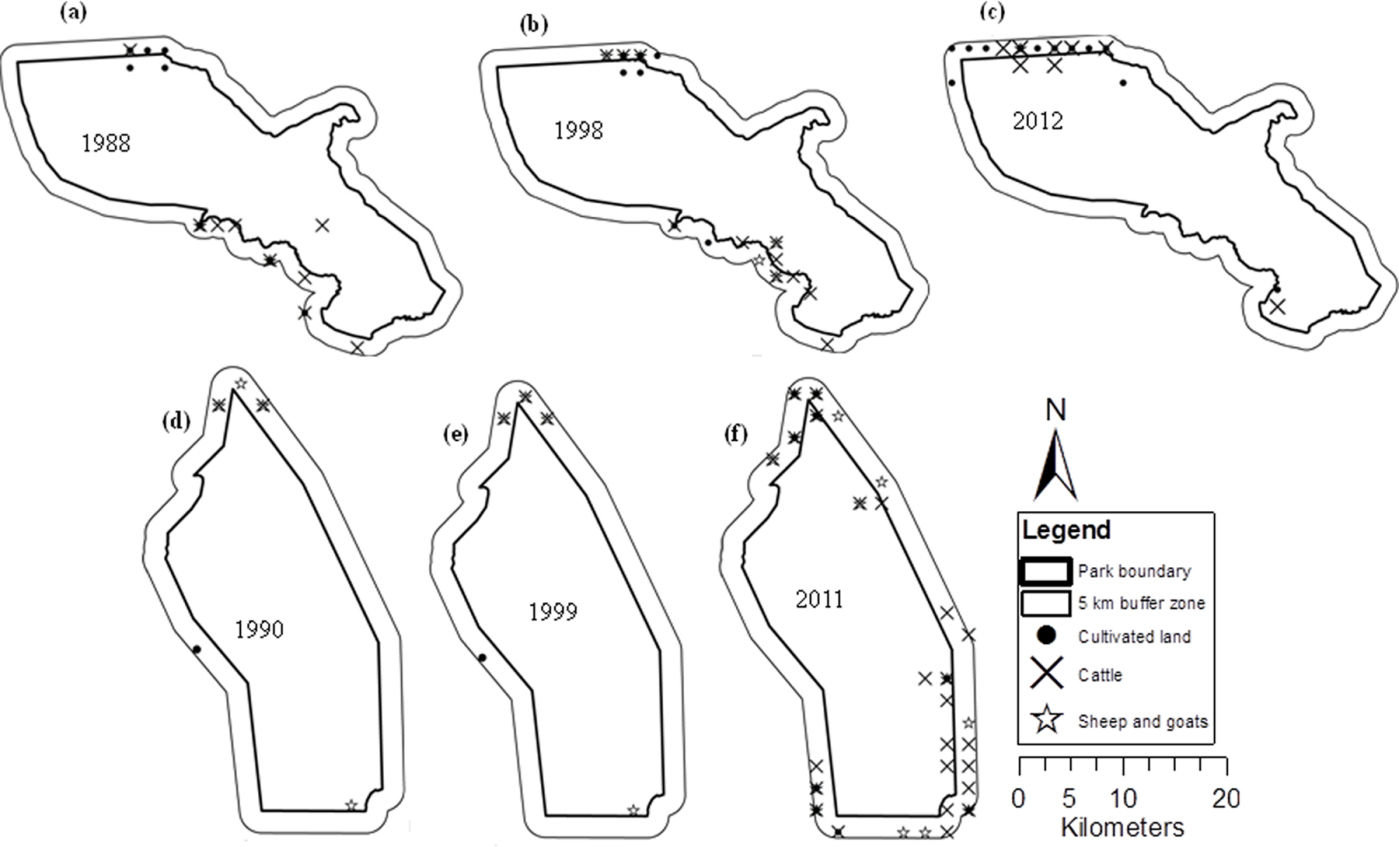
Distribution of human activities (cultivation and livestock keeping) at KNP (a-c) and TNP (d-f) from 1980s to 2010s. [Data source: Tanzania Wildlife Research Institute, 2012].

**Fig 5:**
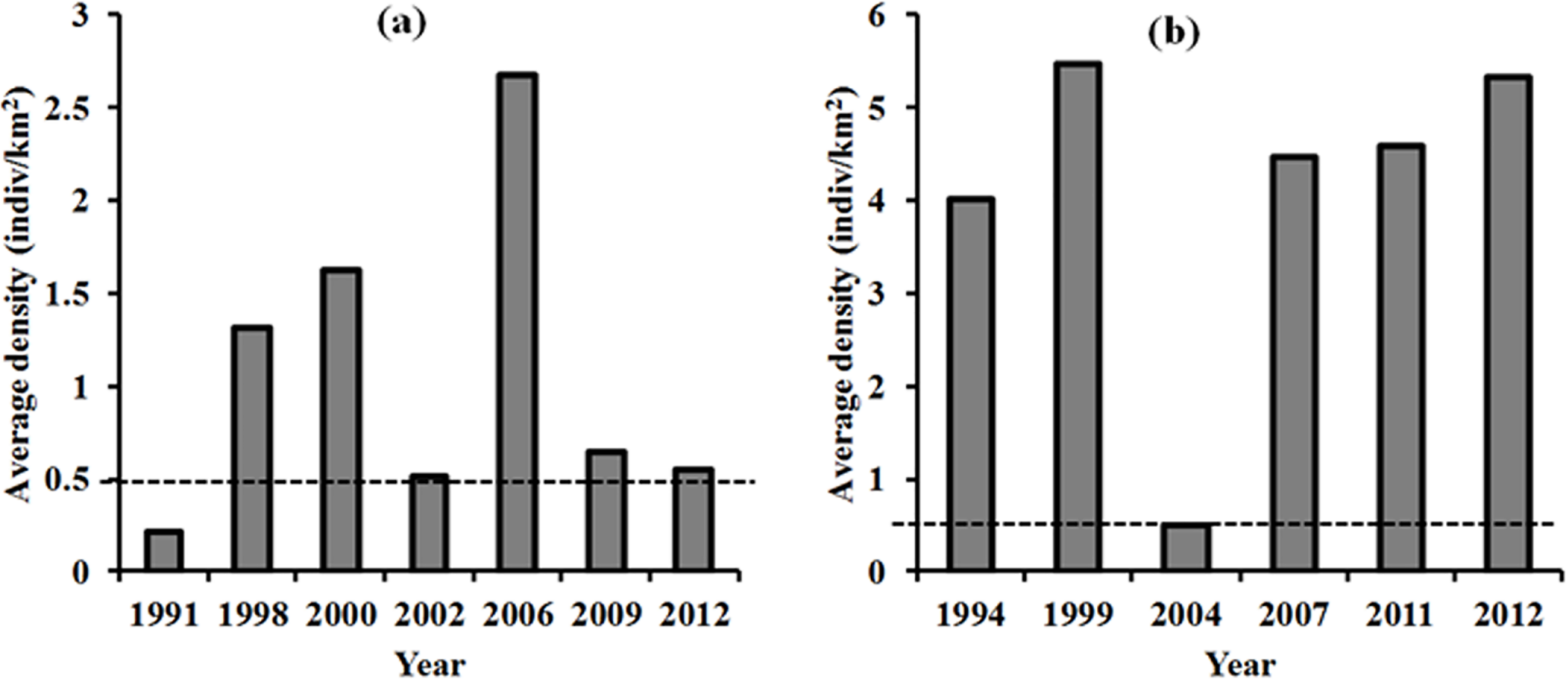
A summary of average elephant densities from 1990s to 2010s recorded in Katavi (a) and Tarangire (b) National Parks. No data were available for 1980s. The dashed line shows the recommended density of 0.5 (individuals/km^2^) [11] to avoid habitat degradation [Data source: Tanzania Wildlife Research Institute, 2012].

For a purpose of discussion of land-cover change detection results, we provide spatial distribution of human activities data (Fig 4) and a bar graph of elephant population density data (Fig 5) that were available from 1990s to 2010s from Tanzania Wildlife Research Institute. Except for 1990s, both datasets for the remaining years were not an exact match of the Landsat imagery data but of a close enough time period to relate changes in land-cover and elephant population.

### Image pre-processing

The six images were geometrically corrected by USGS. The digital numbers (DN) were calibrated to radiance. Then, atmospheric effects were removed using a MODTRAN 5+-based atmospheric correction algorithm known as Fast-Line-of Sight Atmospheric Analysis of Spectral Hypercube (FLAASH) (Exelis Visual Information Solution, Boulder, Colorado) to derive reflectance. The tropical atmospheric model was used for the images with visibility set to 100 km.

### Training data for the 2009 and 2011 Landsat images

Training sites were collected for ten land-cover classes (barren land, cropland, closed shrubland, open shrubland, grassland, savannah, swamp, built-up/natural vegetation mosaic, water bodies, woody savannah) during the preliminary ground survey in December 2011, based on the land-cover definitions used by the International Geosphere-Biosphere Program (IGBP) [42]. Each land-cover class was photographed and a field guide was developed (S1 Table, S1 Fig). Training sites included a total of 59 polygons (431 pixels) for TNP and 54 polygons (351 pixels) for KNP, all collected in the field in June and July 2012. The sampling sites were located approximately 150 m from roadside, and waypoints recorded using Garmin *GPSmap* 62s, which has an accuracy of ± 3.7 m. The vegetation type at each site was entered into the GPS, and a series of photographs taken to aid in data interpretation.

Although ground truth data should be from the same time frame as the remotely sensed image to prevent bias, this is often not possible. One alternative is to use images for the same time of the year with higher spatial resolution [40]. Images such as SPOT and Geoeye which have spatial resolutions of 20 m × 20 m and 1.36 m × 1.36 m respectively, are freely available on Google Earth. Training sites for classifying the 2009 (TNP) and 2011 (KNP) Landsat TM images were obtained using higher resolution images of SPOT and Geoeye from Google Earth. The field data obtained in 2012 aided in the interpretation of the SPOT and Geoeye images, and served as a guide to locate the training sites.

The 2012 locations of each TNP field polygon were visually checked with the SPOT or Geoeye images, depending on availability, for the 2009 image. We suspected that only 31 (84 pixels) of the 59 polygons (431 pixels) drawn in 2012 had the same land-cover type as in 2009. These included polygons that represented woody savannah, closed shrubland, open shrubland, and grassland. Some parts of savannah and swamp were obscured by shadow and clouds or had different land-cover. Therefore, 25 additional sample polygons (285 pixels) for savannah, barren land, water, and swamp were located and selected using SPOT and Geoeye images for 2009, increasing the total training sites to 56 polygons (369 pixels). Through visual inspection 9 polygons (114 pixels) for cloud and shadows were delineated on the 2009 Landsat image, increasing the number of training sites to 67 polygons (493 pixels) which represented 10 land-cover classes. No cropland sample location was found on SPOT or Geoeye photos for the 2009 TNP image.

Following the procedures described above, 38 (234 pixels) out of the 54 polygons (351 pixels) obtained for the KNP in 2012 had the same land-cover types as in 2011. These 38 polygons were located in barren land, closed shrubland, cropland, open shrubland, grassland, savannah, swamp, water bodies, and woody savannah. The locations observed as swamp and water in 2012 were barren and vegetated in 2011, respectively. Therefore, 9 sample polygons (36 pixels) were defined in 2011 SPOT and Geoeye images to represent swamps (5 polygon, 23 pixels) and water (4 polygons, 13 pixels). The total number of training sites was 47 polygons (270 pixels).

The 493 pixels for TNP and 270 pixels for KNP collected using the above validation method were used as training data for classification of the 2009 and 2011 Landsat images, respectively. We used Maximum Likelihood Classification (MLC), for which the minimum number of training pixels required to perform MLC is *N*+1 per class, where *N* represents the number of wavebands. Six bands were used (bands 1 to 5, and 7), yielding a minimum number of pixels per class of 7. Only land cover classes with >10 pixels were used as training data, which included barren land, grassland, open shrubland, closed shrubland, savannah, woody savannah, water bodies, swamp, cloud, and shadow. Cropland and built-up natural vegetation mosaic in TNP had <10 pixels and thus were omitted. Omitting these two land-cover classes was considered negligible as the built-up natural vegetation mosaic was likely to be classified as woody savannah, because most buildings were surrounded by big trees, and cropland was likely to be classified as barren, savannah, or grassland.

An exploratory analysis determined statistical separation between the classes using the Jefferies-Matushita (J-M) distance in order to identify and correct overlapping spectral classes. Values ranged from 1.80 to 2.00 for spectral class pairs, indicating that the pairs were sufficiently separable to classify the 2009 Landsat image. As for the 2011 Landsat image, the J-M distance values ranged from 0.984 to 1.999. Woody savannah vs. natural vegetation mosaic, and savannah vs. cropland had J-M values of 0.984 and 1.287, respectively. A J-M value of below 1.0 may indicate that the class spectral pair is inseparable, while a value of below 1.9 indicates that the class pairs have low separability [43]. Therefore, these two pairs were merged, and the separability for the remaining spectral pairs ranged from 1.5 to 2.0, with 39 polygons (201 pixels) considered separable for classifying the 2011 Landsat image.

### Assessment of classification accuracy of the 2009 and 2011 Landsat TM images

The 2009 Landsat TM image was classified using the MLC Classifier with the 493 training pixels. A random sample of 1,500 points was generated inside the TNP and within the 5 km buffer zone and overlaid on the SPOT and/or GeoEye images in Google Earth. Only 1.5% (22 points) fell on SPOT/Geoeye images of 2009. Some of the remaining 98.5% fell on SPOT/Geoeye images for years other than 2009 and therefore were left out. A total of 22 polygons comprising 1,116 pixels was obtained, which covered seven land-cover classes, including bare land, savannah, grassland, open and closed shrublands, woody savannah, and swamp. Through visual inspection of the Landsat image, 11 additional polygons comprising 217 pixels were directly delineated to represent water, cloud, and shadow. A total of 1,333 testing pixels were used to assess the accuracy of the MLC-classified map of the 2009 TM image using a confusion matrix [44].

The 2011 Landsat TM image was classified using the MLC Classifier with the 270 training pixels. The same procedures used for accuracy assessment of the 2009 TM Landsat image were followed and a total of 32 random polygons comprising 1,211 pixels were obtained that represented eight land-cover classes (bare land, closed shrubland, grassland, open shrubland, savannah, swamp, water, and woody savannah). Therefore, a total of 1,211 testing pixels were used to assess the accuracy of the MLC-classified map for the 2011 TM image using a confusion matrix.

### Classification of Landsat TM image of 1984, 1988 and ETM+ of 1999

The classified maps for 2009 (TNP) and 2011 (KNP) were used as a reference for classification of the 1999, 1988, and 1984 Landsat images because training data for these historical images could not be obtained. Land-cover classes that existed on the 2009 and 2011 Landsat images were assumed to have existed in the 1999 and 1988 (TNP), and 1999 and 1984 (KNP) Landsat images, respectively, based upon similar historical classification approaches [31], [45].

All of the 2009 training pixels, excluding clouds and shadows, were overlaid on the 1999 ETM+ and the 1988 TM image in TNP. The locations for each of the 2009 training pixels were visually examined for land-cover changes using a combination of colour composites of bands 4, 3, 2 (Near Infrared, Red and Green respectively) and 7, 4, 2 (Shortwave Infrared, Near Infrared and Green). These colour composites enabled identification of areas with different vegetation types, in terms of colour and texture. The 4, 3, 2 combination makes vegetation appear as red tones, with brighter reds indicating dense or more growing vegetation, bare soils appearing as white to brown depending on moisture and organic matter, and clear waters appearing as dark blue and shallow waters as lighter blue. Where changes occurred, the pixels were deleted. For example, in 2009, the number of training pixels for water was 63. On the 1988 and 1999 images, six pixels from two polygons were vegetated and therefore were deleted from both images, leaving 57 pixels (4 polygons) for water. A total of 347 pixels (53 polygons) and 370 pixels (54 polygons) were obtained for 1988 and 1999, respectively, and these were used to classify the corresponding images using the MLC. The classification of the 1984 and 1999 Landsat images for KNP followed the same procedures as were used in classifying the 1988 and 1999 in TNP. A total of 137 and 189 training pixels were obtained for 1999 and 1984, respectively, and were used to classify the corresponding images using the MLC.

### Land-cover change detection

Various techniques are used to detect land-cover changes [46–48] and we used the post classification comparison technique. This technique compares independently produced classified images and provides detailed information of land-cover change, including the amount of change, location and the nature of change [46–48]. Although the use of post classification provides a complete matrix of the nature of changes, the accuracy of results may be affected by possible misclassification and registration errors [47–49]. Incorrect classification increases with number of classes and landscape heterogeneity [49].

On the thematic maps derived from the Landsat TM and ETM^+^ for 1984, 1988, 1999, 2009, and 2011, the amount of land-cover and percentages of area for each class, inside and outside the respective parks (TNP and KNP), were calculated and the amount of change determined. Each land-cover class was coded in ArcGIS for each year and a total of six transition matrices were established for both parks using the spatial analyst-*Tabulation Area Tool*. Specifically the six matrices were 1988 vs. 1999, 1999 vs. 2009, and 1988 vs. 2009 for TNP; and 1984 vs. 1999, 1999 vs. 2011and 1984 vs. 2011 for KNP.

### Statistical analysis/decision criterion for determining a change on land cover that is detrimental to wildlife conservation

The sample size for Landsat images used in change detection was insufficient to perform a statistical test to determine the significance of changes in the types of land cover. Therefore, we used a threshold of ≥50% change from the initial time period to determine a substantial change in land-cover that would be potentially detrimental to wildlife conservation, and only land-cover classes with such substantial changes are discussed.

For rainfall, we used a general linear model (GLM) to test for temporal change, from the 1980s to the 2010s, in each park. Because our main research question was to test if rainfall had changed over time, the year variable was treated as a quantitative rather than categorical variable and park was treated as categorical. Wherever the results were significant (*P* ≤ 0.05) for the year variable or the interaction of year and park, estimates of the slopes were obtained using the ESTIMATE statement in the PROC GLM of the SAS system [50] to determine the general trend of change. Results are presented as mean ± error of margin, unless otherwise noted.

Annual elephant density data are provided to visualize the population in relation to the recommended carrying capacity in a preserve and the detected changes in the types of land-cover.

## Results

### Thematic maps derived from Landsat images and accuracy assessment

Thematic maps with 8 land-cover classes (Fig 2) were derived from 2009 and 2011 Landsat TM for TNP and KNP, respectively, with an overall classification accuracy of 86.8% and kappa coefficient of 0.85 (TNP) and 84.6% and 0.80 (KNP) (Tables 1 and 2). Except for water in the KNP, 62.5% to 100% of sample pixels representing all other land-cover classes in the TNP and KNP maps were correctly identified. As for water in KNP, only 33% of sample pixels were correctly identified and this is because it was misidentified as swamp (Table 2). The chances that map users find all land-cover classes (including water) on the ground ranged from 69% to 99% (Tables 1 and 2).

**Table 1:** Confusion matrix of the classification map derived from the 2009 Landsat TM image of the TNP and adjacent areas. [Abbreviation: % User acc. = % User accuracy].

Thematic classes	Reference data											
	Bare land	Cloud	Grassland	Savannah	Shadow	Swamp	Water	Open shr.	Closed shr.	Woody savannah	Row Total	% User Acc.
**Bare land**	**83**	0	0	0	0	0	0	1	0	0	84	98.81
**Cloud**	0	**70**	0	0	0	0	0	0	0	0	70	100
**Grassland**	11	0	**14**1	1	0	0	1	5	1	2	162	87.04
**Savannah**	4	0	0	**66**	0	0	0	0	0	0	70	94.29
**Shadow**	0	0	0	0	**90**	0	0	0	0	0	90	100
**Swamp**	0	0	0	0	0	**55**	0	0	4	3	62	88.71
**Water**	0	0	0	0	0	0	**56**	0	0	0	56	100
**Open shr.**	0	0	12	4	0	0	0	**185**	68	0	269	68.77
**Closed shr.**	0	0	2	0	0	0	0	1	**221**	7	231	95.67
**Woody savannah**	0	0	41	0	0	0	0	2	6	**190**	239	79.5
**Column Total**	98	70	196	71	90	55	57	194	300	202	1333	
**% Producer Accuracy**	84.69	100	71.94	92.96	100	100	98.25	95.36	73.67	94.06		

Overall accuracy = 86.7967%; Kappa Coefficient (K-hat) = 0.8479.

**Table 2:** Confusion matrix of the classification map derived from the 2011 Landsat TM image of the KNP and adjacent areas. [Abbreviation: % User acc. = % User accuracy].

Thematic classes	Reference data									
	Barren land	Closed shr.	Grassland	Open shr.	Savannah	Swamp	Water	Woody savannah	Row Total	% User Acc.
**Bare land**	**89**	0	0	0	0	4	0	0	**93**	95.7
**Closed shr.**	0	**104**	0	1	0	14	0	1	**120**	86.67
**Grassland**	0	0	**126**	0	30	0	0	21	**177**	71.19
**Open shr.**	0	2	0	**5**	0	0	0	0	**7**	71.43
**Savannah**	0	5	14	0	**150**	2	0	33	**204**	73.53
**Swamp**	0	7	0	0	9	**154**	8	2	**180**	85.56
**Water**	0	0	0	0	0	1	**4**	0	**5**	80
**Woody savannah**	6	1	23	2	0	1	0	**392**	**425**	92.24
**Column Total**	95	119	163	8	189	176	12	449	1211	95.7
**% Producer Accuracy**	93.68	87.39	77.3	62.5	79.37	87.5	33.33	87.31		

Overall accuracy = 84.6%, and Kappa coefficient = 0.804.

### Change detection results

Except for woody savannah in KNP and closed shrubland and water in TNP, at least 50% of area covered by all other types of land-cover at TNP and KNP, both inside and outside, changed to other classes in each of the three transitions, the 1980s to 1990s, 1990s to 2010s, and 1980s to 2010s (Tables 3–6 and S2A–S2H Tables). The bare or less vegetated land, open shrubland, grassland, savannah and swamps had ≥ 50% of their 1980s coverage converted or transformed into other types of land-cover by 2010s, both inside the parks and on the adjoining lands (Tables 3–8). As for woody savannah (inside and outside KNP) (Tables 5 and 6 and S2E–S2H), closed shrubland and water (only outside TNP) (Table 4 and S2C and S2D Table), 60% to 100% of their 1980s coverage remained stable through the 2010s. On the other hand the closed shrubland inside TNP was very dynamic with less than 50% of its initial coverage remaining unchanged throughout the study period (Table 3 and S2A and S2B Table).

**Table 3:** Land-cover change (km^2^) inside TNP from 1988 to 2009. The rows and the columns present land-cover classes for 1988 and 2009 respectively. Change (km^2^) in land-cover classes between the years is shown in the last two rows of the table. The diagonal values (bolded) show the amount of cover (km^2^) that remained stable over the 21-year period while the off diagonal values show the amount that was changed to another class.

1988	2009													
	Bare land	Closed shr.	Grassland	Open shr.	Savannah	Swamp	Woody savannah	Water	Shadow	Cloud	Total (km^2^) 1988	Total (km^2^) 2009	(2009–1988 (km^2^)	(2009–1988 (%)
**Bare land**	**0.6**	0.0	3.1	0.1	1.6	0.2	0.2	0.0	0.0	0.2	6.1	58.5	52.4	860.3
**Closed shr.**	12.0	**161.7**	159.3	70.1	1.5	162.9	36.4	0.4	9.4	16.6	630.4	456.7	-173.6	-27.5
**Grassland**	21.3	107.6	**364.8**	89.0	22.1	160.2	72.1	1.2	12.0	25.3	875.8	910.8	35.0	4.0
**Open shr.**	8.8	72.9	73.8	**65.4**	2.8	23.6	38.0	0.0	2.3	6.5	294.1	301.0	7.0	2.4
**Savannah**	4.4	3.4	25.5	7.6	**12.9**	1.7	8.1	0.0	0.3	1.0	64.8	46.0	-18.8	-28.9
**Swamp**	4.7	42.7	72.8	24.3	0.2	**72.1**	16.1	0.2	4.6	7.7	245.3	502.2	256.9	104.7
**Woody sav.**	6.6	68.5	211.6	44.5	4.8	81.7	**51.5**	1.2	7.2	15.8	493.4	222.3	-271.1	-54.9
**Water**											0.0	3.2	3.2	
**Shadow**											0.0	35.9	35.9	
**Cloud**											0.0	73.2	73.2	
											**2609.9**	**2609.9**	**0.0**	

**Table 4:** Land-cover change (km^2^) outside TNP from 1988 to 2009. The rows and the columns present land-cover classes for 1988 and 2009, respectively. Change (km^2^) in land-cover classes between the years is shown in the last two rows of the table. The diagonal values (bolded) show the amount of cover (km^2^) that remained stable over the 21-year period while the off diagonal values show the amount that was changed to another class.

1988	2009													
	Bare land	Closed shr.	Grassland	Open shr.	Savannah	Swamp	Woody savannah	Water	Shadow	Cloud	Total (km^2^) 1988	Total (km^2^) 2009	(2009–1988) (km^2^)	(2009–1988) (%)
**Bare land**	**0.6**	0.0	1.6	0.0	1.1	0.0	0.2	0.0	0.0	0.0	3.5	45.2	41.7	1184.3
**Closed shr.**	8.8	**181.0**	28.9	28.6	2.6	65.5	20.6	0.2	4.2	5.8	346.2	410.7	64.5	18.6
**Grassland**	18.1	80.5	**162.3**	30.6	31.6	69.5	37.2	6.6	5.7	11.7	453.7	282.9	-170.9	-37.7
**Open shr.**	6.3	72.2	14.9	**14.0**	1.0	8.1	14.8	0.0	0.9	2.0	134.2	83.8	-50.4	-37.6
**Savannah**	3.6	1.5	17.5	1.7	**16.0**	1.2	9.1	0.0	0.3	1.6	52.6	56.3	3.7	7.0
**Swamp**	3.9	43.8	9.9	3.2	0.9	**34.8**	7.6	0.4	1.6	3.2	109.3	200.9	91.6	83.8
**Woody sav.**	3.9	31.6	47.7	5.7	3.2	21.8	**10.1**	10.7	1.1	4.0	139.6	40.1	-99.5	-71.3
**Water**	0.0	0.0	0.0	0.0	0.0	0.0	**0.0**	**22.1**	0.0	0.0	22.1	99.5	77.4	349.6
**Shadow**												13.7	13.7	
**Cloud**												28.3	28.3	
**Total**											**1261.3**	**1261.3**	**0.0**	

**Table 5:** Land-cover change (km^2^) inside the KNP from 1984 to 2011. The rows and the columns present land-cover classes for 1984 and 2011 respectively. The total change in land-cover classes between the years is presented in the last two columns. The diagonal values (bolded) show the amount of cover that remained stable over the 27-year period, while the off diagonal values show the amount of cover that was converted to another class. Cloud and shadow are not included in the matrix.

1984	2011													
	Bare land	Closed shr.	Grassland	Open shr.	Savannah	Swamp	Woody savannah	Water	Shadow	Cloud	Total (km^2^) 1984	Total (km^2^) 2011	(2011–1984) (km^2^)	(2011–1984) (%)
**Bare land**	**16.9**	7.4	15.9	2.0	40.7	1.7	40.4	0.0	0.0	0.0	125.1	173.4	48.4	38.7
**Closed shr.**	18.5	**38.9**	45.9	4.4	141.5	17.9	252.9	0.1	0.0	0.0	520.2	222.5	-297.7	-57.2
**Grassland**	22.9	73.3	**117.6**	2.5	629.5	41.9	541.7	0.5	0.0	0.0	1430.0	418.6	-1011.4	-70.7
**Open shr.**	16.8	18.6	47.2	**3.9**	153.7	6.2	104.1	0.0	0.0	0.0	350.5	24.5	-326.0	-93.0
**Savannah**	23.5	29.1	94.1	2.1	**281.8**	12.5	195.3	0.1	0.0	0.0	638.6	1563.7	925.1	144.9
**Swamp**	31.8	9.0	10.8	0.7	79.9	**21.3**	58.5	0.3	0.0	0.0	212.2	133.3	-78.9	-37.2
**Woody sav.**	39.2	42.3	76.4	8.0	182.2	26.1	**476.0**	0.7	0.0	0.0	851.0	1700.3	849.4	99.8
**Water**	0.1	0.1	0.3	0.0	1.4	0.7	0.7	**0.1**	0.0	0.0	3.5	1.9	-1.6	-45.6
**Shadow**	1.9	2.0	4.8	0.4	27.1	2.4	14.9	0.0	0.0	0.0	53.5	0.0	-53.5	-100.0
**Cloud**	1.7	1.9	5.4	0.5	26.0	2.4	15.9	0.0	0.0	0.0	53.8	0.0	-53.8	-100.0
**Total**											**4238.2**	**4238.2**	**0.0**	

**Table 6:** Land-cover change (km^2^) outside the KNP from 1984 to 2011. The rows and the columns present land-cover classes for 1984 and 2011 respectively. The total change in land-cover classes between the years is presented in the last two columns. The diagonal values (bolded) show the amount of cover that remained stable over the 27-year period, while the off diagonal values show the amount of cover that was converted to another class. Cloud and shadow are not included in the matrix.

1984	2011													
	Bare land	Closed shr.	Grassland	Open shr.	Savannah	Swamp	Woody savannah	Water	Shadow	Cloud	Total (km^2^) 1984	Total (km^2^) 2011	(2011–1984) (km^2^)	(2011–1984) (%)
**Bare land**	**17.1**	4.7	3.7	1.5	10.7	1.2	15.2	0.0	0.0	0.0	54.1	193.3	139.2	257.3
**Closed shr.**	40.8	**19.4**	16.6	8.0	54.6	10.6	99.2	0.0	0.0	0.0	249.3	108.1	-141.1	-56.6
**Grassland**	26.2	30.3	**30.9**	6.5	183.9	19.5	295.8	0.5	0.0	0.0	593.6	125.7	-467.9	-78.8
**Open shr.**	14.8	7.0	11.1	**2.5**	30.4	1.8	27.2	0.0	0.0	0.0	94.8	32.7	-62.1	-65.5
**Savannah**	14.0	10.3	19.9	2.0	**58.2**	2.8	53.8	0.1	0.0	0.0	161.0	525.6	364.6	226.4
**Swamp**	21.5	4.5	4.5	1.3	49.5	**8.0**	34.4	0.1	0.0	0.0	123.5	65.2	-58.3	-47.2
**Woody sav.**	56.3	28.9	28.5	9.7	86.3	15.1	**310.7**	0.2	0.0	0.0	535.7	862.7	327.1	61.1
**Water**	0.0	0.2	0.7	0.0	2.6	0.8	1.5	0.0	0.0	0.0	5.9	0.9	-5.0	-84.0
**Shadow**	1.8	1.2	2.1	0.5	11.0	2.1	9.6	**0.0**	0.0	0.0	28.3	0.0	-28.3	-100.0
**Cloud**	0.7	1.8	7.7	0.6	38.5	3.3	15.4	0.0	0.0	0.0	68.1	0.0	-68.1	-100.0
**Total**											**1914.3**	**1914.3**	**0.0**	

**Table 7:** Major conversion/transformation *from-to* classes inside and on the lands adjoining the Tarangire National Park from 1988 to 2009.

Inside: From class in 1988	To class in 2009	Area (km^2^)	% Coverage	Outside: From class in 1988	To class in 2009	Area (km^2^)	% Coverage
Closed shrubland	Bare land	12,0	20,6	Closed shr.	Savannah	141.5	27.2
Grassland	Bare land	21,3	36,4	Closed shr.	Woody savannah	252.9	48.6
Closed shrubland	Swamp	162,9	32,4	Grassland	Savannah	629.5	44.0
Grassland	Swamp	160,2	31,9	Grassland	Woody savannah	541.7	37.9
Woody savannah	Closed shrubland	68,5	13,9	Open shr.	Savannah	153.7	43.9
Woody savannah	Grassland	211,6	42,9	Open shr.	Woody savannah	104.1	29.7
				Woody savannah	Savannah	182.2	11.7
				Savannah	Woody savannah	195.3	11.5

**Table 8:** Major conversion/transformation *from-to* classes inside and on the lands adjoining the Katavi National Park from 1984 to 2011.

Inside: From	To class	Area	%	Outside: From	To class	Area	%
class in 1984	in 2011	(km^2^)	Coverage	class in 1984	in 2011	(km^2^)	Coverage
Closed shr.	Savannah	141.5	27.2	Closed shr.	Bareland	40.8	21.1
Closed shr.	Woody savannah	252.9	48.6	Grassland	Bareland	26.2	13.6
Grassland	Savannah	629.5	44.0	Swamp	Bareland	21.5	11.1
Grassland	Woody savannah	541.7	37.9	Woody sav.	Bareland	56.3	29.2
Open shr.	Savannah	153.7	43.9	Closed shr.	Savannah	54.6	21.9
Open shr.	Woody savannah	104.1	29.7	Closed shr.	Woody savannah	99.2	39.8
woody savannah	Savannah	182.2	11.7	Grassland	Savannah	183.9	31.0
Savannah	Woody savannah	195.3	11.5	Grassland	Woody savannah	295.8	49.8
				Open shr.	Savannah	30.4	32.1
				Open shr.	Woody savannah	27.2	28.7

Overall, areas covered by bare or less vegetated land and swamps, both inside and outside TNP, expanded by 80% to 1000% between 1988 to 2009, while woody savannah decreased by 55% to 70% at both park locations (Tables 3 and 4), and areas under water expanded outside the park by 350% (Table 4). The areas covered by woody savannah and savannah at KNP expanded, both inside and outside the park, by 60 to 230% between 1984 and 2011, while shrublands, grassland, swamps and water decreased by 50 to 100% (Tables 5 and 6). The bare or less vegetated land remained relatively unchanged inside the park (Table 5) but increased by 260% outside the park (Tables 6 and 8).

### Change of rainfall over time

No significant change in annual rainfall was found in both KNP and TNP over the 15 and 17 year periods, respectively (Fig 3), even though rainfall records in TNP for the past 10 years from 2000 to 2009 (except for 2005 and 2006) were consistently below the annual average. Rainfall in 2005 and 2006 were equal 605 mm and 642.9 mm, respectively (Fig 3).

## Discussion

The substantial decrease of woody savannah in TNP from 1988 to 2009 was due to its conversion into grassland, while the increase of bare or less vegetated land was due to a conversion from grassland, open shrubland, savannah and woody savannah (Tables 3, 4 and 7). Previous research in the TNP ecosystem found declines of woody vegetation and grassland resulting from their conversion into cultivated farms and settlements [20], [31]. Although we were unable to identify and assess cultivated or cropland covers, observations from aerial survey data collected by TAWIRI between 1980 and 2011 show an expansion in land used for cultivation and livestock grazing within 5 km inside and outside the park boundary (Fig 4). Additionally, observations based on 2012 Geoeye and SPOT high-resolution photos show the continual existence of farm plots inside and outside TNP (S2 Fig). Hence, human encroachment may be the primary cause for the decrease and increase of woody savannah and bare or less vegetated land, respectively, in TNP. The secondary cause of landscape change may be the high elephant densities in the TNP, which during the years of this study (1994–1999 and 2007) were 10 times higher than the recommended limit of 0.5 individuals/km^2^ (Fig 5) [11]. When elephant density exceeds the reserve’s carrying capacity, and their movements are restricted to within the parks, they degrade vegetation cover [11] through overgrazing and/or over browsing, trampling and knocking down trees [51], and accelerate soil erosion [17]. In arid savannah, woody vegetation has been found to respond negatively to high elephant densities [52,53]. For instance, in Sweetwaters game reserve in Kenya, elephants densities of 1.2 individuals/km^2^ were responsible for the loss of 40% of trees between 1998 and 2001, while the remaining loss was attributed to rhino damage and drought [54].

Woody vegetation was reported to have declined between 1971 and 1996 in TNP, due to the severe drought that occurred in 1993, and possibly an earlier drought, from 1991 to 1992 [55]. During the 1993 drought, total annual rainfall was well below the 655 mm annual average (Fig 3). The amount of annual rainfall received in TNP between 2007 and 2009 was even lower than that recorded in 1993 (Fig 3). Furthermore, the 2009 rainfall level corresponds to the amount which supports only a low amount of woody cover [3]. Therefore, the decreased amount of woody savannah in 2009 was probably due to low amount of rainfall. However, despite the lower than normal rainfalls observed in 2007–2009, there was no significant decrease in rainfall between 1988 and 2009. As a result, it is possible that the lower amounts of green vegetation in 2009 could have caused a higher level of misclassification than expected.

Grassland and closed shrubland contributed to the increase in swamp coverage at TNP and was parallel to the increase in amount of water outside the park (Tables 4 and 6). The increase in swamp coverage may have as well resulted from the extremely high El niño rains of 1997–1998. Notably, the heavy rains began in 1996 and lasted until 1999 with highest levels falling in 1997–1998 (Fig 3). Such high levels of rainfall might have recharged the swamps and therefore resulted in the observed increase in swamp coverage. In addition, the increase of water and swamps at TNP may have contributed by the implementation of the national wildlife policy of 1998 (amended in 2007). The policy states that: “Wildlife and wetlands are natural resources of great biological, economical, environmental cleaning, climate ameliorating, water and soil conservation, and nutritional values that must be conserved. It can be used indefinitely if properly managed” [56]. On the other hand, if we exclude the El Niño and the implementation of the wildlife policy, but consider the below average amount of rainfall from 2007 to 2009 (Fig 3) and the observed human activities outside the TNP (Fig 4 and S2 Fig), the increase of swamps in TNP could possibly be a result of errors during the classification of the Landsat images.

**Fig 6:**
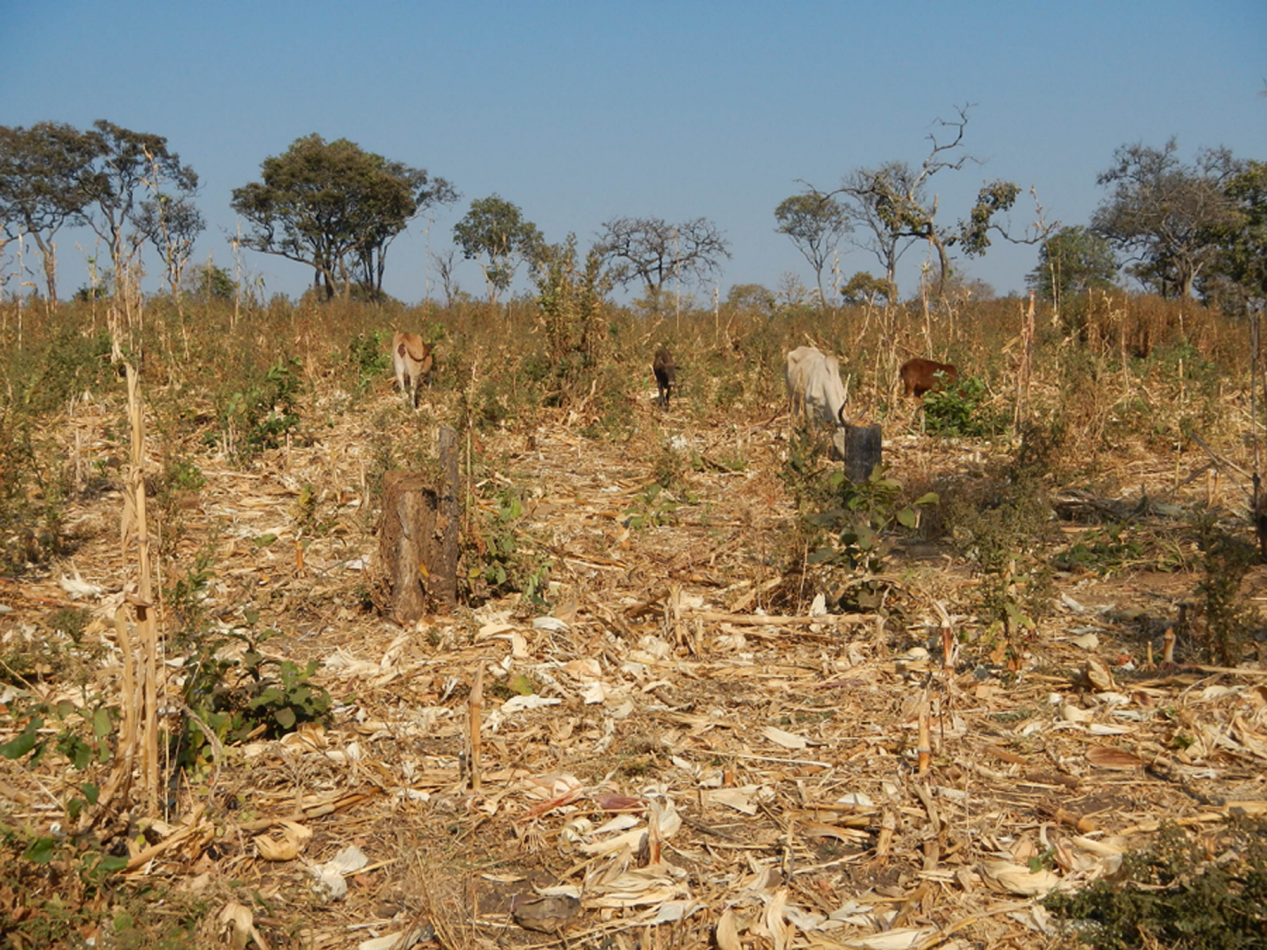
Cropland with scattered trees resembling savannah type of land-cover at Katavi areas.

At KNP, grassland and shrubland decreased from 1984 to 2011, both inside and outside the park, and were replaced by savannah and woody savannah (Tables 5, 6 and 8 and Fig 2). This change in shrubland was probably contributed by the extension of the park in 1999 to cover a large area, which was not previously protected (Figs 1 and 2). The KNP receives rainfall above the annual average of 955 mm, a level that promotes tree recruitment into adult age classes and closed canopy in the absence of disturbances such as frequent fires and herbivory [3,4], [9]. The increase in the size of KNP and the subsequent implementation of the wildlife policy of 1999 are with no doubt the reason for the observed increase of savannah and woody savannah. One of the strategy which might have contributed to the increase of the latter land-cover classes was the establishment of Community Conservation Schemes under a Katavi-Rukwa Development program that was funded by Germany Agency for Technical Cooperation (GTZ-Wildlife program) [57]. This program was aimed at improving wildlife conservation by promoting sustainable use of wildlife resource. Our study did not determine causal effect of fires or herbivory, and therefore we were unable to provide their contribution to present changes on land cover. The savannah class was merged with croplands during the satellite image analysis, as the two were spectrally inseparable (see Methods). Most croplands observed around KNP have scattered trees (Fig 6), a possible reason for their overlap with savannah. Cultivation and livestock have been observed mostly outside the park (Fig 4), although a few farm plots also occur inside the park (S3 Fig). Therefore, the conclusion that savannah increased at KNP should be treated with caution due to the location of the park. For example the increase of savannah from 1984 to 2011 on the northern and/or North-west KNP, within 5km inside and outside of the park boundary, most likely represented an increase in croplands or a mixture of savannah and croplands. Observations made on 2013 Google Earth images also support the existence of cultivated land inside the KNP (S3 Fig).

In contrast with our results, Banda and others [24], who assessed the structure and composition of woody vegetation in the Katavi ecosystem in 2000 and 2003, reported declining densities of woody trees, at a higher rate inside than outside the KNP, due to over browsing by large herbivores. The increase in canopy trees reported in the present study likely resulted from their recovery after 13 years of park protection in the expansion area (Figs 1 and 2). Banda and others [24] might not have detected this recovery because their assessment was conducted only five years after the park expansion.

Our results suggest that woody savannah decreased in TNP and increased in KNP. These findings are similar to those previous reports [34] which indicated that the amount of vegetation greenness (NDVI index) during the dry season across Tanzania, between 1982 and 1994, decreased in northern Tanzania, where TNP is located, and increased in the other parts of the country, including the KNP ecosystem. Huete [58] correlated NDVI index with various canopy covers and found that the greener and denser the cover type was, the higher the NDVI was. Taking into account these two studies, the changes in the amount of greenness, declining inside and outside the TNP and increasing in the KNP area, imply that vegetation condition is worsening in TNP and improving in KNP.

Wildlife species utilize different types of vegetation cover in different seasons [59,60]. For example, some species of herbivores such as elephants, prefer open woodland, shrubland and grassland during the wet season [59] and closed woodlands during the dry season [17], [59], [61]. The decline of either type of land cover is therefore of concern for their continual survival. For example, the population of wildebeest in TNP declined from 40,000 to 5,000 between 1984 and 2000, due to habitat degradation and conversion of woodland and grassland into farmland and settlement [31]. In the Mara Region of Kenya, resident wildebeest population declined from 119,000 to 22,000 within a period of 20 years from 1977 to 1997 due to conversion of their wet season habitat into agriculture [62]. Likewise, in the same region the population of resident and migratory species of wildebeest and zebra declined both in the wet and dry season between 1977 and 2009, primarily due to expansion of human population [63] while remaining unchanged in the adjoining Serengeti ecosystem where they migrate from [64]. In Mara Region of Kenya, wildebeest (*Connochaetes taurinus*), Grant gazelle (*Gazella granti*), Thomson gazelle (*Gazella thomsonii*) and zebra (*Equus burchellii*) populations declined by 60% between 1975 and 2007 due to conversion of grasslands to agriculture [21]. Skarpe and others [65] reported an increase in numbers of buffalo (*Syncerus caffer*), impala (*Aepyceros melampus*), and greater kudu (*Tragelaphus strepsiceros*) with increase of shrubland. The substantial decline of woody savannah in TNP, closed shrubland, grassland and swamps in KNP therefore are of concern for conservation and may result in food shortage for wildlife populations.

## Limitations

The accuracy of our results may be limited by several factors, including the inconsistent dates for the Landsat scenes used in the analysis, particularly in KNP where images were acquired in June, July and September, even though they all were from the same dry season. Specifically, the timing of the imagery may have introduced some errors during the classification of the Landsat images. False classification of change is known to increase with the number of classes and landscape heterogeneity [49]. Future analyses of Landsat imagery data might consider using fewer classes to see if it improves the current results. The resolution (30 m) of the satellite data (Landsat TM and ETM+) used in the analysis may be considered low for certain types of land cover and land use classifications because it was not possible to separate savanna from cropland/cultivated land during the classification process. Other confounding variables, such as fires, may also have contributed to the land-cover changes [4], [5], [7–10], [12,13], [16], [66].

## Conclusion

Over the past 27 years, woody savannah declined in TNP, primarily due to human encroachment, resulting in the increase of bare or less vegetated land inside and outside the park. On the other hand, the woodlands (woody savannah and savannah) at KNP increased, both inside and outside the park, replacing the shrublands and grassland. This increase was a result of the expansion of the park in 1999. The swamps increased inside and outside TNP, while at KNP the change was not substantial. The decrease of woody savannah and the increase of bare or less vegetated land at TNP pose a conservation threat to wildlife species. Therefore, we recommend management action to minimize human activities encroaching on the parks and confining wildlife within parks boundaries hence leading to degradation of their habitats.

## Supporting information

S1 Fig Land-cover photos produced as field guide after the ground preliminary survey in 2011. (TIF)

S2 Fig Cultivated lands inside and outside the Tarangire National Park. The green line shows park boundary, white lines show the farmed plots lettered A to C inside, and D outside the park [Source Google Earth 2011 to 2013].
(TIF)

S3 Fig Cultivated lands inside the Katavi National Park. The green line shows park boundary and white lines show the farmed plots inside the park [Source Google Earth 2011].
(TIF)

S1 Table Definition of land-cover classes as identified at the Tarangire and Katavi National Parks (adapted and modified from Friedl and others [42]. (DOC)

S2 Table **S2A–H Tables**. Land-cover change (km^2^) inside and outside Tarangire National Park from 1988 to 1999 (A and C), and from 1999 to 2009 (B and D) and; Katavi National Park from 1984 to 1999 (E and G), and from 1999 to 2011 (F and H). The rows and the columns present land-cover classes for 1988 and 1999 respectively. Change (km^2^) in land-cover classes between the years is shown in the last two rows of the table. The diagonal values (bolded) show the amount of cover (km^2^) that remained unchanged over the 10-year period while the off diagonal values show the amount that was changed to another class.
(DOC)
