# Supplementary figures and images for: Assessing multi-decadal land-cover – land-use change in two wildlife protected areas in Tanzania using Landsat imagery
Source: PLoS One. 2017 Sep 28;12(9):e0185468. doi: 10.1371/journal.pone.0185468 (PMC5619789; doi:10.1371/journal.pone.0185468)

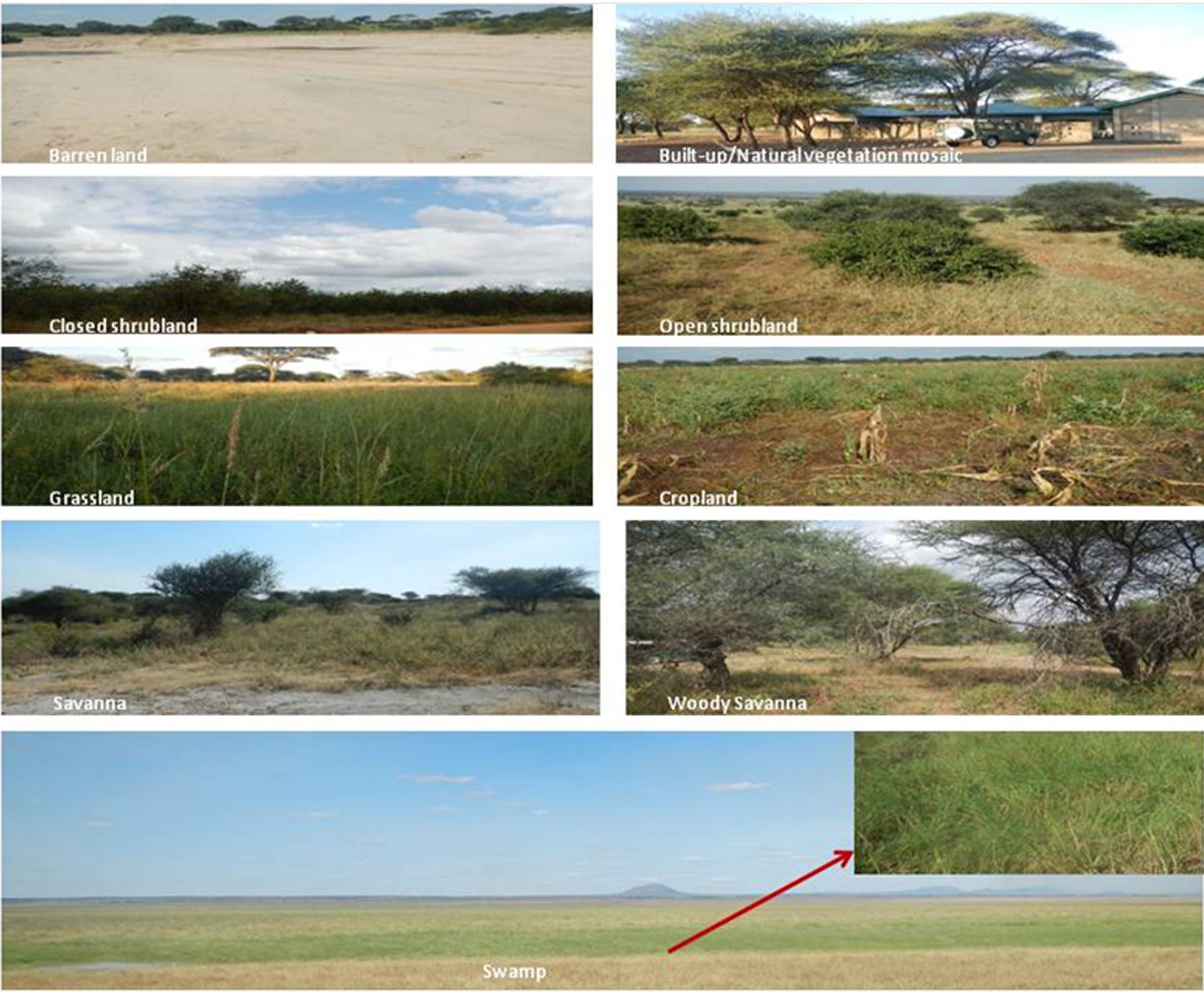

Supplement: S1 Fig — (TIF) [file pone.0185468.s001.tif]

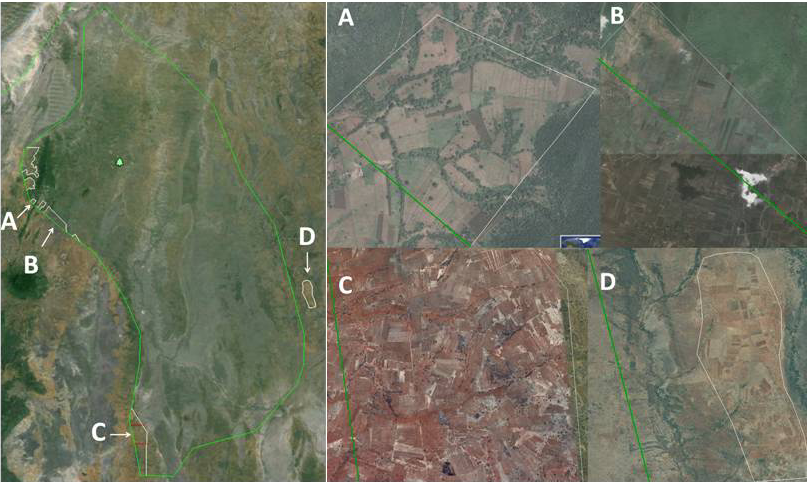

Supplement: S2 Fig — The green line shows park boundary, white lines show the farmed plots lettered A to C inside, and D outside the park [Source Google Earth 2011 to 2013]. (TIF) [file pone.0185468.s002.tif]

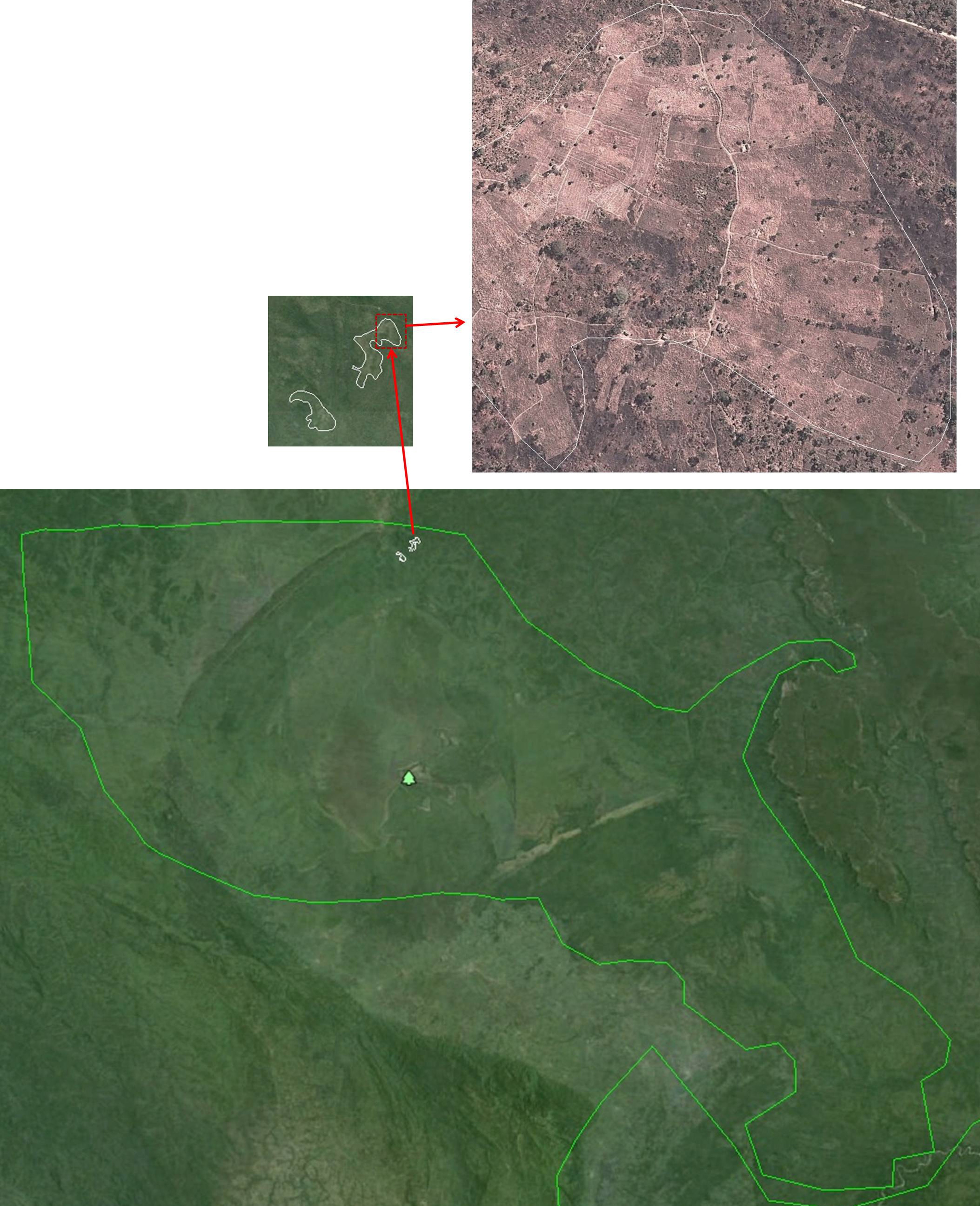

Supplement: S3 Fig — The green line shows park boundary and white lines show the farmed plots inside the park [Source Google Earth 2011]. (TIF) [file pone.0185468.s003.tif]
