# Supplementary material for: Assessing multi-decadal land-cover – land-use change in two wildlife protected areas in Tanzania using Landsat imagery
Source: PLoS One. 2017 Sep 28;12(9):e0185468. doi: 10.1371/journal.pone.0185468 (PMC5619789; doi:10.1371/journal.pone.0185468)
Supplement: S1 Table — (DOC) [file pone.0185468.s004.doc]

S1 Table. Definition of land cover classes as identified at the TNP and KNP (adapted and modified from Friedl and others [40].

| **Land cover type** | **Definition** |
| --- | --- |
| Bare land or less vegetated land | Lands with exposed soils, sand or rocks, with no more than 10% vegetation cover at any time of the year. |
| Built up/natural vegetation mosaic | Lands with houses at least 40%, and 60% composed of natural vegetation (trees, shrubs grass, forbs), bare land (rock, soils or sand) (modified definition). |
| Closed shrubland | Land with woody vegetation (evergreen or deciduous) < 2 meters tall and with shrub canopy cover >60%. |
| Cropland | Lands covered with temporary crops followed by harvest and bare-soil period e.g. single and multiple cropping systems. Perennial woody crops are classified as the appropriate shrubland cover type. |
| Grassland | Lands with variety of grasses and forbs. Woody vegetation cover (trees and shrubs) is less than 10%. |
| Open shrubland | Lands with woody vegetation (evergreen or deciduous) < 2 meters tall and with shrub canopy cover between 10-60%. |
| Savannah | Lands with grasses, forbs, and other understory systems and with tree canopy cover between 10 and 30% The tree canopy cover height exceed 2 meters. |
| Swamp | Lands with mixture of water and grasses and forbs, or woody vegetation. The water often moving perceptibly, supporting low vegetation (e.g. sedges), reeds and woody vegetation. |
| Water bodies | Lakes, streams, rivers and water holes (boreholes). |
| Woody savannah | Lands with grasses, forbs and other understory systems, with canopy cover between 30 and 60%. The trees height exceeds 2m and can be deciduous or evergreen. |
