# Supplementary material for: Assessing multi-decadal land-cover – land-use change in two wildlife protected areas in Tanzania using Landsat imagery
Source: PLoS One. 2017 Sep 28;12(9):e0185468. doi: 10.1371/journal.pone.0185468 (PMC5619789; doi:10.1371/journal.pone.0185468)
Supplement: S2 Table — S2A–H Tables. Land-cover change (km2) inside and outside Tarangire National Park from 1988 to 1999 (A and C), and from 1999 to 2009 (B and D) and; Katavi National Park from 1984 to 1999 (E and G), and from 1999 to 2011 (F and H). The rows and the columns present land-cover classes for 1988 and 1999 respectively. Change (km2) in land-cover classes between the years is shown in the last two rows of the table. The diagonal values (bolded) show the amount of cover (km2) that remained unchanged over the 10-year period while the off diagonal values show the amount that was changed to another class. (DOC) [file pone.0185468.s005.doc]

S2 Table A. Land cover change (km^2^) inside TNP from 1988 to 1999. The rows and the columns present land cover classes for 1988 and 1999 respectively. Change (km^2^) in land cover classes between the years is shown in the last two rows of the table. The diagonal values (bolded) show the amount of cover (km^2^) that remained unchanged over the 10-year period while the off diagonal values show the amount that was changed to another class.

| **1999**  **1988** | **Bare land** | **Closed shr.** | **Grassland** | **Open shr.** | **Savannah** | **Swamp** | **Woody savannah** | **Total (km^2^)**  **1988** | **Total (km^2^)**  **1999** | **(1999 - 1988) (km^2^)** | **(1999 - 1988)**  **(%)** |
| --- | --- | --- | --- | --- | --- | --- | --- | --- | --- | --- | --- |
| **Bare land** | **0.8** | 0.1 | 2.8 | 0.3 | 2.0 | 0.0 | 0.2 | 6.1 | 28.6 | 22.5 | 368.9 |
| **Closed shr.** | 2.2 | **158.2** | 52.7 | 138.4 | 40.6 | 94.8 | 143.4 | 630.4 | 489.1 | -141.3 | -22.4 |
| **Grassland** | 15.3 | 106.1 | **211.5** | 149.0 | 128.9 | 105.1 | 160.0 | 875.8 | 446.9 | -428.8 | -49.0 |
| **Open shr.** | 0.3 | 72.4 | 16.8 | **94.7** | 36.0 | 25.9 | 48.0 | 294.1 | 496.6 | 202.5 | 68.9 |
| **Savannah** | 2.4 | 4.2 | 18.0 | 9.3 | **24.0** | 1.2 | 5.7 | 64.8 | 308.5 | 243.7 | 376.1 |
| **Swamp** | 0.9 | 50.8 | 26.6 | 34.3 | 11.1 | **75.1** | 46.6 | 245.3 | 367.1 | 121.7 | 49.6 |
| **Woody sav.** | 6.8 | 97.3 | 118.5 | 70.6 | 66.0 | 64.9 | **69.3** | 493.4 | 473.1 | -20.3 | -4.1 |
| **Total** | | | | | | | | **2609.9** | **2609.9** | **0.0** |  |

S2 Table B. Land cover change (km^2^) inside TNP from 1999 to 2009. The rows and the columns present land cover classes for 1999 and 2009, respectively. Change (km^2^) in land cover classes between the years is shown in the last two rows of the table. The diagonal values (bolded) show the amount of cover (km^2^) that remained unchanged over the 10-year period while the off diagonal values show the amount that was changed to another class.

| **2009**  **1999** | **Bare land** | **Closed shr.** | **Grassland** | **Open shr.** | **Savannah** | **Swamp** | **Woody savannah** | **Water** | **Shadow** | **Cloud** | **Total (km^2^)**  **1999** | **Total (km^2^)**  **2009** | | **(2009 -1999) (km^2^)** | | **(2009 - 1999)**  **(%)** | |
| --- | --- | --- | --- | --- | --- | --- | --- | --- | --- | --- | --- | --- | --- | --- | --- | --- | --- |
| **Bare land** | **2.0** | 0.3 | 20.1 | 0.5 | 1.3 | 2.0 | 0.7 | 0.5 | 0.5 | 0.8 | 28.6 | | 58.5 | | 29.9 | | 104.8 |
| **Closed shr.** | 8.7 | **155.4** | 136.9 | 75.6 | 1.9 | 60.3 | 28.7 | 0.1 | 7.8 | 13.8 | 489.1 | | 456.7 | | -32.4 | | -6.6 |
| **Grassland** | 10.5 | 23.7 | **225.8** | 23.8 | 13.8 | 89.3 | 31.7 | 1.9 | 7.5 | 18.9 | 446.9 | | 910.8 | | 463.8 | | 103.8 |
| **Open shr.** | 16.6 | 128.6 | 120.8 | **90.0** | 6.6 | 53.8 | 64.1 | 0.2 | 4.3 | 11.6 | 496.6 | | 301.0 | | -195.5 | | -39.4 |
| **Savannah** | 9.3 | 45.5 | 124.9 | 48.0 | **20.7** | 14.9 | 33.3 | 0.1 | 4.0 | 7.8 | 308.5 | | 46.0 | | -262.5 | | -85.1 |
| **Swamp** | 3.6 | 48.0 | 125.7 | 31.0 | 0.3 | **123.9** | 19.6 | 0.2 | 6.0 | 9.0 | 367.1 | | 502.2 | | 135.1 | | 36.8 |
| **Woody sav.** | 7.9 | 55.3 | 156.7 | 32.1 | 1.4 | 158.1 | **44.3** | 0.3 | 5.8 | 11.3 | 473.1 | | 222.3 | | -250.8 | | -53.0 |
| **Water** | - | - | - | - | - | - | - | - | - | - | - | | 3.2 | | 3.2 | |  |
| **Shadow** | - | - | - | - | - | - | - | - | - | - | 0.0 | | 35.9 | | 35.9 | |  |
| **Cloud** | - | - | - | - | - | - | - | - | - | - | 0.0 | | 73.2 | | 73.2 | |  |
| **Total** | | | | | | | | | | | **2609.9** | | **2609.9** | | **0.0** | |  |

S2 Table C. Land cover change (km^2^) outside TNP from 1988 to 1999. The rows and the columns present land cover classes for 1988 and 1999 respectively. Change (km^2^) in land cover classes between the years is shown in the last two rows of the table. The diagonal values (bolded) show the amount of cover (km^2^) that remained stable over the 10-year period while the off diagonal values show the amount that was changed to another class.

| **1999**    **1988** | **Bare land** | **Closed shr.** | **Grassland** | **Open shr.** | **Savannah** | **Swamp** | **Woody savannah** | **Water** | **Total (km^2^) 1988** | **Total (km^2^) 1999** | **(1999 - 1988) (km^2^)** | **(1999 - 1988)**  **(%)** |
| --- | --- | --- | --- | --- | --- | --- | --- | --- | --- | --- | --- | --- |
| **Bare land** | **0.8** | 0.0 | 1.3 | 0.3 | 0.8 | 0.0 | 0.2 | 0.0 | 3.5 | 28.6 | 25.0 | 710.8 |
| **Closed shr.** | 0.4 | **94.7** | 21.4 | 137.7 | 9.1 | 15.5 | 67.4 | 0.0 | 346.2 | 182.2 | -163.9 | -47.4 |
| **Grassland** | 17.4 | 23.8 | **118.5** | 119.9 | 64.6 | 13.8 | 91.2 | 4.5 | 453.7 | 206.8 | -246.9 | -54.4 |
| **Open shr.** | 0.1 | 22.6 | 5.8 | **76.6** | 4.2 | 4.0 | 20.9 | 0.0 | 134.2 | 398.1 | 263.9 | 196.7 |
| **Savannah** | 5.1 | 1.2 | 13.7 | 6.6 | **22.2** | 0.3 | 3.7 | 0.0 | 52.6 | 112.4 | 59.8 | 113.6 |
| **Swamp** | 0.5 | 24.8 | 9.1 | 27.6 | 2.2 | **11.7** | 33.3 | 0.2 | 109.3 | 50.5 | -58.8 | -53.8 |
| **Woody sav.** | 4.3 | 15.1 | 36.9 | 29.5 | 9.3 | 5.1 | **29.4** | 9.9 | 139.6 | 246.0 | 106.3 | 76.2 |
| **Water** | 0.0 | 0.0 | 0.0 | 0.0 | 0.0 | 0.0 | 0.0 | **22.1** | 22.1 | 36.8 | 14.6 | 66.1 |
| **Total** | | | | | | | | | **1261.3** | **1261.3** | **0.0** |  |

S2 Table D. Land cover change (km^2^) outside TNP from 1999 to 2009. The rows and the columns present land cover classes for 1999 and 2009, respectively. Change (km^2^) in land cover classes between the years is shown in the last two rows of the table. The diagonal values (bolded) show the amount of cover (km^2^) that remained stable over the 10-year period while the off diagonal values show the amount that was changed to another class.

| **2009**  **1999** | **Bare land** | **Closed shr.** | **Grassland** | **Open shr.** | **Savannah** | **Swamp** | **Woody savannah** | **Water** | **Shadow** | **Cloud** | **Total (km^2^)**  **1999** | **Total (km^2^)**  **2009** | **(2009 -1999) (km2)** | **(2009 - 1999)**  **(%)** |
| --- | --- | --- | --- | --- | --- | --- | --- | --- | --- | --- | --- | --- | --- | --- |
| **Bare land** | **3.5** | 0.6 | 14.9 | 0.3 | 4.9 | 1.2 | 2.3 | 0.1 | 0.1 | 0.7 | 28.6 | 45.2 | 16.7 | 58.4 |
| **Closed shr.** | 3.3 | **106.3** | 16.2 | 10.6 | 1.4 | 30.3 | 8.4 | 0.4 | 2.8 | 2.5 | 182.2 | 410.7 | 228.4 | 125.3 |
| **Grassland** | 8.8 | 20.3 | **93.2** | 8.2 | 15.9 | 35.5 | 13.6 | 1.5 | 2.0 | 7.7 | 206.8 | 282.9 | 76.1 | 36.8 |
| **Open shr.** | 17.1 | 195.3 | 53.9 | **43.6** | 7.6 | 30.2 | 40.6 | 0.2 | 3.0 | 6.7 | 398.1 | 83.8 | -314.3 | -79.0 |
| **Savannah** | 4.3 | 9.0 | 49.3 | 4.7 | **24.0** | 7.3 | 9.6 | 0.0 | 1.0 | 3.2 | 112.4 | 56.3 | -56.1 | -49.9 |
| **Swamp** | 0.9 | 11.3 | 9.5 | 3.3 | 0.2 | **19.0** | 3.0 | 1.0 | 1.0 | 1.4 | 50.5 | 200.9 | 150.4 | 297.7 |
| **Woody sav.** | 7.3 | 67.9 | 45.8 | 13.1 | 2.5 | 76.9 | **22.**0 | 0.6 | 3.8 | 6.1 | 246.0 | 99.5 | -146.5 | -59.5 |
| **Water** | 0.0 | 0.0 | 0.1 | 0.0 | 0.0 | 0.4 | 0.0 | **36.2** | 0.0 | 0.0 | 36.8 | 40.1 | 3.3 | 9.1 |
| **Shadow** | - | - | - | - | - | - | - | - | - | - | 0.0 | 13.7 | 13.7 |  |
| **Cloud** | - | - | - | - | - | - | - | - | - | - | 0.0 | 28.3 | 28.3 |  |
| **Total** | | | | | | | | | | | **1261.3** | **1261.3** | **0.0** |  |

S2 Table E. Land cover change (km^2^) inside the KNP from 1984 to 1999. The rows and the columns present land cover classes for 1984 and 1999 respectively. The total change in land cover classes between the years is presented in the last two columns. The diagonal values (bolded) show the amount of cover that remained stable over the 10-year period, while the off diagonal values show the amount of cover that was converted to another class. Cloud and shadow are not included in the matrix.

| **1999**  **1984** | **Bare**  **land** | **Closed shr.** | **Grassland** | **Open shr.** | **Savannah** | **Swamp** | **Woody savannah** | **Water** | **Shadow** | **Cloud** | **Total (km^2^) 1984** | **Total (km^2^) 1999** | **(1999 - 1984) (km^2^)** | **(1999 - 1984)**  **(%)** |
| --- | --- | --- | --- | --- | --- | --- | --- | --- | --- | --- | --- | --- | --- | --- |
| **Bare land** | **27.2** | 9.6 | 26.9 | 7.2 | 30.5 | 2.0 | 20.0 | 1.7 | 0.0 | 0.0 | 125.1 | 286.4 | 161.4 | 129.0 |
| **Closed shr.** | 20.4 | **88.4** | 227.1 | 17.0 | 42.6 | 5.2 | 112.2 | 7.4 | 0.0 | 0.0 | 520.2 | 582.3 | 62.1 | 11.9 |
| **Grassland** | 90.7 | 217.5 | **609.4** | 36.6 | 240.8 | 13.5 | 196.0 | 25.5 | 0.0 | 0.0 | 1430.0 | 1587.0 | 157.0 | 11.0 |
| **Open shr.** | 24.4 | 23.5 | 116.3 | **14.8** | 110.6 | 2.2 | 54.2 | 4.5 | 0.0 | 0.0 | 350.5 | 132.5 | -218.0 | -62.2 |
| **Savannah** | 49.1 | 46.1 | 236.1 | 20.3 | **184.7** | 4.3 | 88.2 | 9.8 | 0.0 | 0.0 | 638.6 | 770.3 | 131.7 | 20.6 |
| **Swamp** | 11.8 | 25.6 | 57.2 | 8.2 | 33.8 | **30.3** | 34.0 | 11.1 | 0.0 | 0.0 | 212.2 | 66.9 | -145.3 | -68.5 |
| **Woody sav.** | 57.4 | 163.4 | 258.0 | 24.7 | 107.4 | 7.6 | **208.9** | 23.5 | 0.0 | 0.0 | 851.0 | 726.8 | -124.2 | -14.6 |
| **Water** | 0.2 | 0.2 | 1.8 | 0.1 | 0.3 | 0.1 | 0.4 | **0.4** | 0.0 | 0.0 | 3.5 | 86.1 | 82.6 | 2379.7 |
| **Shadow** | 2.2 | 4.2 | 27.1 | 1.6 | 9.2 | 1.0 | 6.9 | 1.2 | 0.0 | 0.0 | 53.5 | 0.0 | -53.5 |  |
| **Cloud** | 3.0 | 3.7 | 27.2 | 1.9 | 10.4 | 0.6 | 6.0 | 1.1 | 0.0 | 0.0 | 53.8 | 0.0 | -53.8 |  |
| **Total** | | | | | | | | | | | **4238.2** | **4238.2** | **0.0** |  |

S2 Table F. Land cover change (km^2^) inside the KNP from 1999 to 2011. The rows and the columns present land cover classes for 1999 and 2011, respectively. The total change in land cover classes between the years is presented in the last two columns. The diagonal values (bolded) show the amount of cover that remained stable over the10-year period, while the off diagonal values show the amount of cover that was converted to another class. Cloud and shadow are not included in the matrix.

| **2011**  **1999** | **Bare land** | **Closed shr.** | **Grassland** | **Open shr.** | **Savannah** | **Swamp** | **Woody savannah** | **Water** | **Total (km^2^) 1999** | **Total (km^2^) 2011** | **(2011 -1999) (km^2^)** | **(2011 -1999) (%)** |
| --- | --- | --- | --- | --- | --- | --- | --- | --- | --- | --- | --- | --- |
| **Bare land** | **33.9** | 11.5 | 37.5 | 2.3 | 135.5 | 5.5 | 60.2 | 0.0 | 286.4 | 173.4 | -113.0 | -39.5 |
| **Closed shr.** | 14.6 | **60.2** | 31.4 | 6.4 | 122.6 | 20.2 | 326.9 | 0.0 | 582.3 | 222.5 | -359.7 | -61.8 |
| **Grassland** | 8.4 | 73.9 | **143.2** | 2.7 | 574.5 | 43.4 | 740.8 | 0.2 | 1587.0 | 418.6 | -1168.4 | -73.6 |
| **Open shr.** | 14.4 | 12.3 | 15.3 | **3.8** | 56.9 | 5.5 | 24.2 | 0.1 | 132.5 | 24.5 | -108.0 | -81.5 |
| **Savannah** | 39.3 | 27.9 | 115.4 | 3.3 | **438.8** | 13.8 | 131.6 | 0.2 | 770.3 | 1563.7 | 793.4 | 103.0 |
| **Swamp** | 27.8 | 0.8 | 2.8 | 0.0 | 17.1 | **6.7** | 11.6 | 0.1 | 66.9 | 133.3 | 66.4 | 99.4 |
| **Woody sav.** | 26.3 | 33.1 | 69.2 | 5.3 | 189.2 | 21.9 | **381.7** | 0.2 | 726.8 | 1700.3 | 973.5 | 133.9 |
| **Water** | 8.7 | 3.0 | 3.7 | 0.7 | 29.1 | 16.2 | 23.4 | **1.1** | 86.1 | 1.9 | -84.2 | -97.8 |
| **Total** | | | | | | | | | **4238.2** | **4238.2** | **0.0** |  |

S2 Table G. Land cover change (km^2^) outside the KNP from 1984 to 1999. The rows and the columns present land cover classes for 1984 and 1999 respectively. The total change in land cover classes between the years is presented in the last two columns. The diagonal values (bolded) show the amount of cover that remained stable over the 10-year period, while the off diagonal values show the amount of cover that was converted to another class. Cloud and shadow are not included in the matrix.

| **1999**  **1984** | **Bare**  **land** | **Closed shr.** | **Grassland** | **Open shr.** | **Savannah** | **Swamp** | **Woody savannah** | **Water** | **Shadow** | **Cloud** | **Total**  **(km^2^)**  **1984** | **Total**  **(km^2^)**  **1999** | **(1999 - 1984) (km^2^)** | **(1999 - 1984)**  **(%)** |
| --- | --- | --- | --- | --- | --- | --- | --- | --- | --- | --- | --- | --- | --- | --- |
| **Bare land** | **6.1** | 6.2 | 11.2 | 5.8 | 9.1 | 0.5 | 11.1 | 4.0 | 0.0 | 0.0 | 54.1 | 92.1 | 38.0 | 70.3 |
| **Closed shr.** | 9.7 | **47.0** | 75.2 | 17.7 | 20.9 | 1.5 | 65.1 | 12.1 | 0.0 | 0.0 | 249.3 | 344.6 | 95.3 | 38.2 |
| **Grassland** | 27.6 | 135.3 | **214.7** | 9.9 | 55.7 | 0.5 | 137.1 | 12.8 | 0.0 | 0.0 | 593.6 | 628.6 | 35.0 | 5.9 |
| **Open shr.** | 5.7 | 8.5 | 33.0 | **5.5** | 17.8 | 0.2 | 19.6 | 4.4 | 0.0 | 0.0 | 94.8 | 77.3 | -17.4 | -18.4 |
| **Savannah** | 9.5 | 14.5 | 67.9 | 4.6 | **27.5** | 0.1 | 30.9 | 5.8 | 0.0 | 0.0 | 161.0 | 203.1 | 42.1 | 26.2 |
| **Swamp** | 5.7 | 13.7 | 45.7 | 8.3 | 22.2 | **1.9** | 20.4 | 5.7 | 0.0 | 0.0 | 123.5 | 7.9 | -115.6 | -93.6 |
| **Woody sav.** | 24.3 | 109.9 | 122.6 | 23.0 | 35.9 | 3.1 | **199.7** | 17.2 | 0.0 | 0.0 | 535.7 | 496.0 | -39.6 | -7.4 |
| **Water** | 0.1 | 0.6 | 3.9 | 0.3 | 0.3 | 0.0 | 0.6 | **0.1** | 0.0 | 0.0 | 5.9 | 64.5 | 58.6 | 993.1 |
| **Shadow** | 0.6 | 3.4 | 15.4 | 0.6 | 3.2 | 0.0 | 3.5 | 1.6 | 0.0 | 0.0 | 28.3 | 0.0 | -28.3 |  |
| **Cloud** | 2.8 | 5.4 | 39.0 | 1.6 | 10.5 | 0.0 | 8.0 | 0.8 | 0.0 | 0.0 | 68.1 | 0.0 | -68.1 |  |
| **Total** | | | | | | | | | | | **1914.3** | **1914.3** | **0.0** |  |

S2 Table H. Land cover change (km^2^) outside the KNP from 1999 to 2011. The rows and the columns present land cover classes for 1999 and 2011, respectively. The total change in land cover classes between the years is presented in the last two columns. The diagonal values (bolded) show the amount of cover that remained stable over the 10-year period, while the off diagonal values show the amount of cover that was converted to another class. Cloud and shadow are not included in the matrix.

| **2011**  **1999** | **Bare land** | **Closed shr.** | **Grassland** | **Open shr.** | **Savannah** | **Swamp** | **Woody savannah** | **Water** | **Total (km^2^)**  **1999** | **Total (km^2^)**  **2011** | **(2011-1999) (km^2^)** | **(2011 -1999)**  **(%)** |
| --- | --- | --- | --- | --- | --- | --- | --- | --- | --- | --- | --- | --- |
| **Bare land** | **13.49** | 4.73 | 9.78 | 1.53 | 35.82 | 2.37 | 24.35 | 0.02 | 92.1 | 193.3 | 101.2 | 109.9 |
| **Closed shr.** | 19.89 | **23.17** | 11.73 | 8.72 | 51.00 | 9.10 | 220.96 | 0.05 | 344.6 | 108.1 | -236.5 | -68.6 |
| **Grassland** | 23.91 | 39.68 | **55.52** | 8.48 | 241.45 | 26.08 | 232.86 | 0.65 | 628.6 | 125.7 | -502.9 | -80.0 |
| **Open shr.** | 33.92 | 5.54 | 3.18 | **2.34** | 12.58 | 4.74 | 15.02 | 0.01 | 77.3 | 32.7 | -44.7 | -57.8 |
| **Savannah** | 37.42 | 7.41 | 20.04 | 2.99 | **98.84** | 3.54 | 32.85 | 0.07 | 203.1 | 525.6 | 322.5 | 158.7 |
| **Swamp** | 3.89 | 0.27 | 0.05 | 0.05 | 0.22 | **0.65** | 2.78 | 0.00 | 7.9 | 65.2 | 57.3 | 723.9 |
| **Woody sav.** | 30.55 | 23.68 | 24.38 | 6.67 | 78.18 | 16.49 | **315.96** | 0.12 | 496.0 | 862.7 | 366.7 | 73.9 |
| **Water** | 30.18 | 3.66 | 1.02 | 1.87 | 7.52 | 2.27 | 17.97 | **0.02** | 64.5 | 0.95 | -63.6 | -98.5 |
| **Total** | | | | | | | | | **1914.3** | **1914.3** | **0.0** |  |
